# Socioeconomic inequalities and the economic burden of dental caries: a longitudinal simulation analysis across forty countries

**DOI:** 10.1186/s12889-026-28363-4

**Published:** 2026-07-24

**Authors:** Neeladri Verma, Gerard Dunleavy, Amanda Stucke, Shivangi Jain, Julia Maciel de Rodrigues, Joao Hofmeister, Moritz Kebschull, Iain Chapple, Nicola West, Nigel Pitts

**Affiliations:** 1 Economist Impact, Hong Kong, Hong Kong; 2 Economist Impact, London, UK; 3 Economist Impact, São Paulo, Brazil; 4 NIHR Biomedical Research Centre Birmingham, The University of Birmingham, Birmingham, UK; 5 Birmingham Community Healthcare NHS Trust, Birmingham, UK; 6 Division of Periodontics, Section of Oral, Diagnostic and Rehabilitation Sciences, College of Dental Medicine, Columbia University, New York, NY USA; 7 Periodontal Research Group, Dentistry, School of Health Sciences, College of Medicine and Health, University of Birmingham, Birmingham, UK; 8 Bristol Dental School, University of Bristol, Bristol, UK; 9 Faculty of Dentistry, Oral & Craniofacial Sciences, King’s College London, London, UK

**Keywords:** Oral health, Dental caries, Inequalities, Inclusivity, Economic burden, Prevention, Non-communicable diseases, Public health, Socioeconomic

## Abstract

**Background:**

In 2024, World Health Organization (WHO) member states endorsed the Bangkok declaration, prioritising oral diseases within universal health coverage and non-communicable disease agendas, and calling for prevention-oriented, primary care-led systems. This study aimed to: (1) explore the healthcare costs of managing dental caries between the ages of 12 and 65 years across income quintiles in 40 countries, and (2) estimate the potential reduction in direct costs from non-targeted and targeted oral health-promoting interventions.

**Methods:**

A cohort simulation model was developed to estimate the direct costs of dental caries over time for different income quintiles across 40 countries. National-level DMFT (dentine threshold) data, the relative likelihood of receiving an intervention (such as a restorative procedure, tooth extraction and replacement), and clinically guided assumptions were used to populate the model. Private treatment costs in each country were used as a proxy to estimate the direct costs of dental caries. A hypothetical group of upstream and downstream preventive interventions were applied either uniformly across all income groups to reduce caries progression rates by 30% or in a “levelling-up” fashion with the greatest gains seen in the lowest income group.

**Results:**

The population-level direct costs of caries from 12 to 65 years of age varied between US$317.8 million in Slovenia to US$182.6 billion in China. The highest per-person costs were in Switzerland at US$51,932 and the lowest in Jordan at US$6,339. The per-person direct costs were highest in the lowest income quintile 40 countries. Inadequate preventative oral care for lower income groups results in 50% higher total treatment costs. With the uniform application of preventive measures across all income quintiles, the greatest reduction in per-person costs for caries management was seen in the lowest income quintile across all countries. With a levelling-up approach, cost reductions in the lowest income group ranged from US$4,083 in Vietnam to US$43,106 in Switzerland.

**Conclusion:**

Lower income groups experience a greater health and economic burden from dental caries. Early, targeted preventive interventions, such as twice daily brushing with fluoridated toothpaste, water fluoridation, oral health education, and school programs, within a life-course, equity-driven framework, can reduce costs, mitigate disease burden, and advance global oral health equity.

**Supplementary Information:**

The online version contains supplementary material available at https://doi.org/10.1186/s12889-026-28363-4.

## Background

In November 2024, WHO Member States endorsed the *Bangkok declaration: No Health Without Oral Health*, elevating oral diseases within the universal health coverage and non-communicable disease agendas and calling for strengthened, primary care-led, prevention-oriented systems [1]. The Declaration emphasised equity, sustainability and accountability, reflecting the growing recognition of the wide health and societal impact of oral diseases.

Untreated dental caries is the most prevalent health condition worldwide, affecting an estimated 2.5 billion people. It causes pain, infection, diminished quality of life, and, in severe cases, tooth loss and systemic complications. Beyond its health implications, the economic burden of dental caries is considerable, encompassing both direct treatment costs and indirect productivity losses. Yet robust, comparable economic evidence to guide policy, investment and prioritisation across countries remains limited [2, 3]. Embedding these methods more systematically is essential if the commitments of the Bangkok Declaration are to be translated into budgetary decisions and health service redesign.

Against this policy backdrop, in 2024, an international team of oral health clinicians, policy experts, and health economists developed an initial proof-of-concept economic modelling framework to estimate (1) the direct costs of managing dental caries among individuals aged 12 to 65 years, and (2) the potential reductions in these costs achievable through universal and targeted oral health promotion interventions across different socioeconomic groups in six countries—Brazil, France, Germany, Indonesia, Italy, and the United Kingdom [4]. As part of the Health Inclusivity Index research initiative, this framework was subsequently enhanced and extended to all 40 Health Inclusivity Index countries—spanning low-, middle-, and high-income settings across Africa, Asia, Europe, Latin America, the Middle East, and North America, enabling the first-ever global-scale quantification of oral health inequities’ economic burden. Our previous research found that individuals in the lowest income quintiles consistently bear much higher per-person costs for dental caries and encounter systemic barriers to accessing both preventive and treatment services, underscoring persistent intranational inequities in oral health burden. Expanding the analysis to a broader set of countries is essential, as the health burden of tooth decay continues to rise while comprehensive assessments of its economic impact remain scarce. This lack of economic evidence hampers policymakers’ ability to fully appreciate the scale and urgency of the problem; robust economic analyses are therefore critical to quantifying its costs and guiding timely, evidence-based interventions, with the aim of delivering oral healthcare as part of universal healthcare.

## Methods

**Fig. 1:**
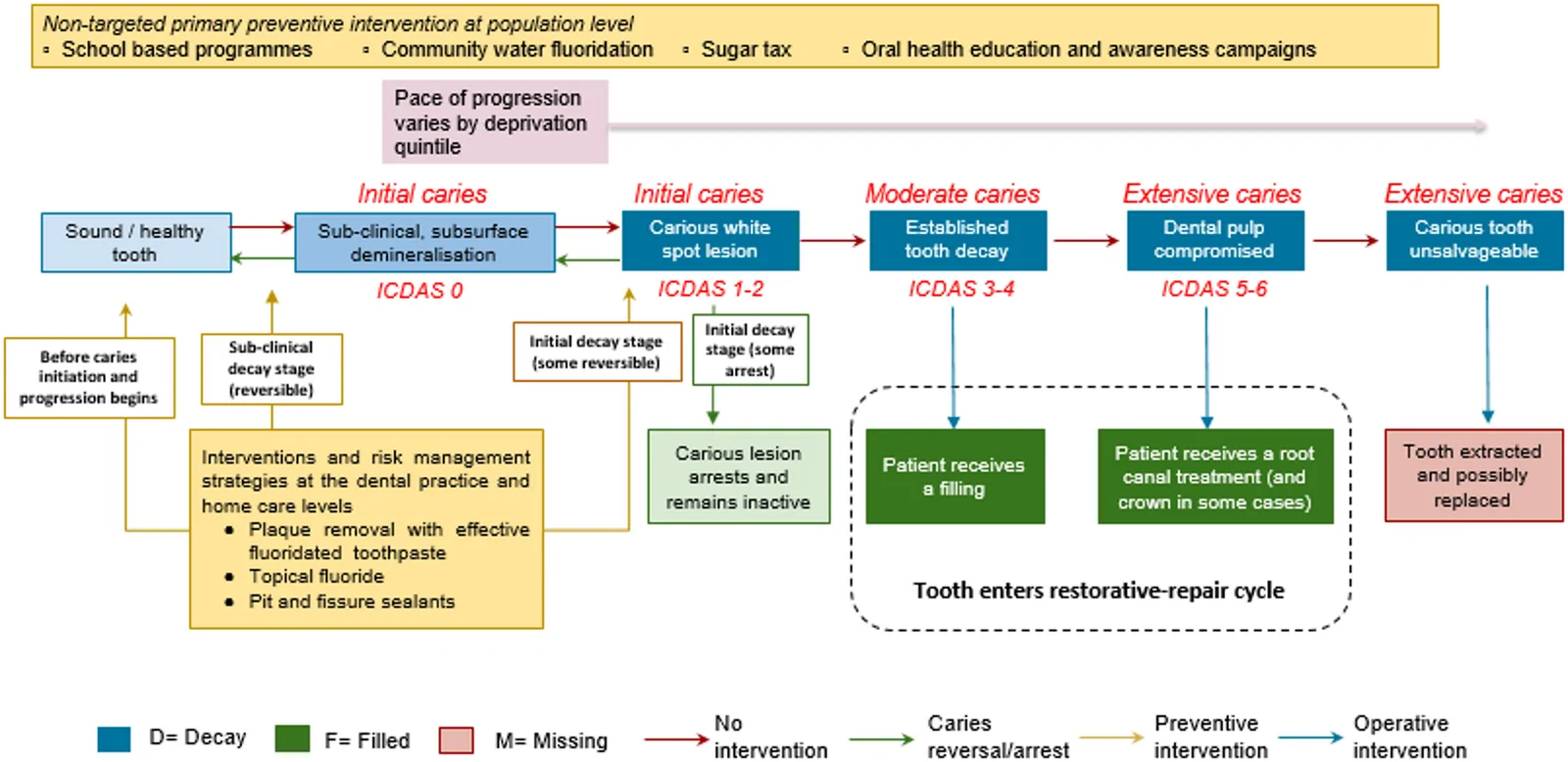
Conceptual framework of dental caries pathway of care

A description of the conceptual framework illustrating the progression of dental caries, together with a detailed account of the modelling approach and steps, has been published elsewhere [4]. In brief, the framework traces the natural history of dental caries from a healthy tooth through successive stages—initial white spot lesion, cavitated caries, pulp involvement, and ultimately an unsalvageable tooth. Preventive interventions, such as brushing with fluoridated toothpaste, topical fluoride application, fissure sealants, and consumption of fluoridated products, are mapped to early and intermediate stages. Once cavitation occurs, management shifts to restorative care and, in advanced cases, root canal therapy or extraction and replacement as appropriate. Figure 1 provides a visual summary of this staged approach and corresponding interventions.

Figure 1 illustrates the caries progression pathway and shows how each stage of the process can be mapped to ICDAS categories for reporting (i.e., which stage of the caries continuum falls under which ICDAS category). The figure is not intended to imply that every lesion at a given ICDAS category necessarily leads to a single clinical outcome (for example, we are not stating that all ICDAS 5–6 lesions represent pulp involvement). In Fig. 1, we also link the ICDAS/tooth stage to the DMF classification and associated treatment options in the second column. Thus, the figure uses ICDAS to stage lesion severity/activity, and then connects that staging to the practical reporting/DMF framework used for treatment modelling and cost implications.

**Fig. 2:**
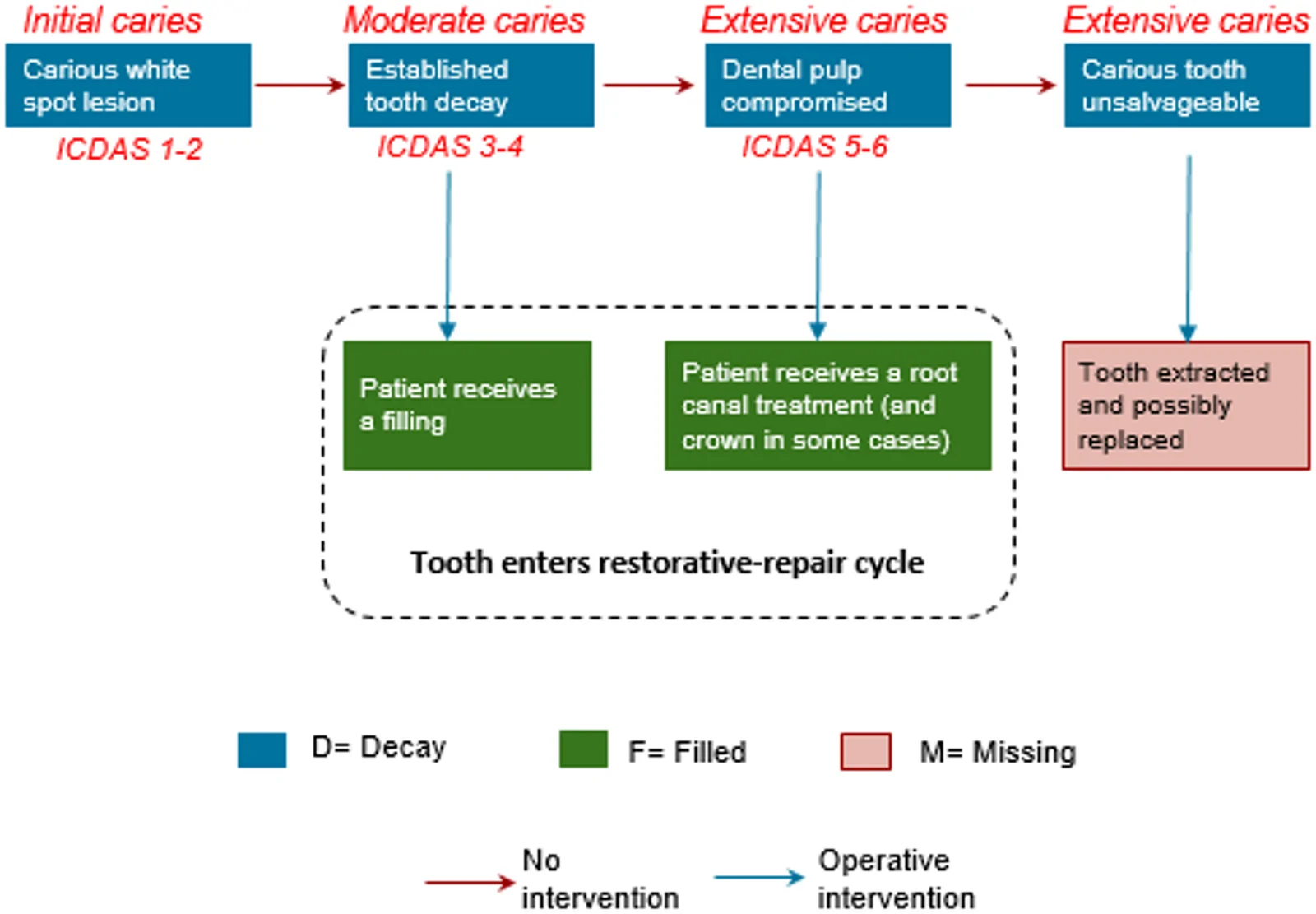
Framework for analysis

Based on this conceptual framework, we developed a simplified approach to the dental caries clinical care pathway, based on available data, to enable an estimation of the direct costs of managing dental caries (Fig. 2). [5–7]. We adhered to the Consolidated Health Economic Evaluation Reporting Standards (CHEERS) reporting guidelines to ensure comprehensive and transparent reporting of our analysis [8].

A detailed overview of the data inputs and methods used in our analysis are available elsewhere [4]. Briefly, national population data were sourced from the World Population Prospects (WPP, 27th edition) [9], with age structures derived from 5-year cohorts and projections accounted for general mortality trends [9, 10]. Cohorts were disaggregated by income quintile, with 20% of the population assigned to each income quintile [11]. DMFT scores for 12-year-olds were obtained from national oral health surveys and modelled across socioeconomic groups using adjustments reflecting estimates of DMFT score variations by income group as well as national income inequality (Gini coefficient) [12, 13]. The DMFT scores by income quintile and country are detailed in Supplementary Table 1 in Supplementary File 1 [14–22]. The assumptions used in the distribution of DMFT progression between age cohorts across income quintiles are detailed in Supplementary Table 2 in Supplementary File 1.

Annual caries progression was derived from systematic review data and applied differentially by income level [23], with further disaggregation following established clinical cohort studies [24]. Estimates of direct treatment costs were based on a triangulation of expert input and online, country-specific cost data. The costs associated with dental caries management per tooth are detailed in Supplementary Table 3 in Supplementary File 1. Given variation in the provision of subsidised care across countries and the lack of information on public-sector healthcare costs, private treatment costs in each country were used as a proxy to estimate the direct costs of dental caries.

Provision rates for root canals, crowns, and tooth replacements across income groups are reported in Supplementary Tables 4 and 5 of Supplementary File 1. In the initial analysis, 30% of individuals in the second most deprived quintile and 60% in the most deprived quintile were assumed not to receive any tooth replacement following extraction [4]. However, for the Health Inclusivity Index analysis, these rates were adjusted to 0% to model the scenario in which each income quintile receives a basic standard of care, including tooth replacement. This approach enabled a more equitable estimation of treatment costs across all groups.

Given substantial differences in price levels, fee structures and healthcare financing arrangements between countries, the absolute cost estimates generated by the model are not intended to serve as precise, directly comparable budget figures. Instead, they provide harmonised, order‑of‑magnitude estimates that allow us to examine within‑country gradients in costs across income quintiles and to explore how these gradients vary across heterogeneous settings. Cross‑country comparisons are therefore used primarily to illustrate the range of economic burden profiles and the consistency of socioeconomic patterns, rather than to rank countries on the basis of absolute expenditure.

### Scenario analysis

Two scenario analyses were performed to assess the decrease in per capita costs between 12 and 65 years of age based on the following interventions:

- Scenario A – Application of universal interventions to deliver a decrease in caries progression rates by 30% across each income quintile. The 30% decrease in dental caries progression rate was considered conservative, given that the majority of dental caries is preventable via a range of effective public health interventions, such as community water fluoridation, salt fluoridation, reduced sugar consumption (via the implementation of, for example, sugar-sweetened beverage [SSB] taxes or enhanced food labelling) and twice daily brushing with fluoridated toothpaste and fluoride varnish application (frequency dependent on risk of caries) [25]. The efficacy of these interventions is detailed in Table 1.
- Scenario B – A ‘levelling-up’, or proportionate universalism approach, with the scale of prevention and management interventions proportional to the degree of need across income quintiles. In this scenario, we applied an annual increment of 0.07 in DMFT across all income quintiles, based on the adjusted progression estimate reported by Hummel et al. [23]. This lower progression rate reflects settings in which preventive interventions are more systematically implemented and is used here as an aspirational benchmark for a “levelling‑up” scenario, rather than as an immediately achievable target in all countries.

Scenario B should be interpreted as an illustrative “levelling‑up” scenario rather than as a forecast. By applying the empirically observed lower annual increment in DMFT associated with greater preventive coverage to all income quintiles, we approximate an upper bound on the cost reductions that could be achieved if disadvantaged groups attained caries progression rates similar to those of better‑served populations. This strong assumption is not meant to reflect current realities, but to highlight the scale of potential gains from ambitious, equity‑focused prevention policies and to show that the largest relative benefits would accrue to lower income groups.

**Table 1:** An overview of effective interventions for caries prevention

Interventions	Efficacy of the interventions
Community water fluoridation	Water fluoridation can prevent: • 35% caries lesions in the primary teeth [25] • 26% caries lesions in permanent teeth [25] • 15% of any new caries lesions (primary disease prevention) [25]
Salt fluoridation	Shown to prevent 50% of new caries lesions [26]
Reduced sugar consumption	• Limiting free sugar intake to ≤ 10% of total energy is linked to a lower risk of dental caries, but does not completely prevent it [27] • Data suggests that a lower intake of free sugars, equivalent to < 5% of total energy, may further reduce the risk of dental caries [27]
SSB taxes	A recent umbrella review concluded that a 20% sugar tax would reduce sugar intake by 18% and 20% in low and middle-income and high-income countries, respectively. The review reported that SSB taxes would result in a positive but modest impact on oral health, reducing dental caries prevalence in children by 2.9% and 2.7% for low and middle-income and high-income countries, respectively, reducing caries counts in adults by 0.03, in both low and middle-income and high-income countries [28].
Fluoridated toothpaste	• Fluoridated toothpaste can prevent 20–30% of caries lesions [25] • Highest fluoride concentrations (2400–2800 ppm) toothpaste can prevent 37% of caries lesions [25] • Over-the-counter fluoride toothpaste (1000–1500 ppm) can prevent 12% of new caries lesions [25]
Fluoride varnish	Shown a 37% decrease in new caries lesions in school-aged children [25]

### Statistical analysis

Student’s t-tests were used to assess whether dental costs in the baseline scenario differed across income quintiles. A two-tailed *p*-value of < 0.05 was considered statistically significant. All analyses were conducted using Stata version 15.0 (StataCorp, College Station, TX, USA).

### Model validation

Consistency checks, face validity assessments and a sensitivity analysis were conducted as part of the model’s internal validation. Consistency checks involved amending data and model parameters such as baseline DMFT data, direct costs and probabilities for tooth health transition states were amended to ensure that the model behaved logically, resulting in the expected changes in outcomes. Face validity was ensured by engaging experts in health economics, epidemiology, and oral health experts to review the model to ensure that the assumptions, data inputs, and overall structure were reasonable and reflected current knowledge. We conducted a sensitivity analyses, adjusting the discount rate from 3.5% to 5%, reflecting the upper limit of discount rates applied in a systematic review of modelling techniques for the economic evaluation of dental caries [29].

## Results

**Fig. 3:**
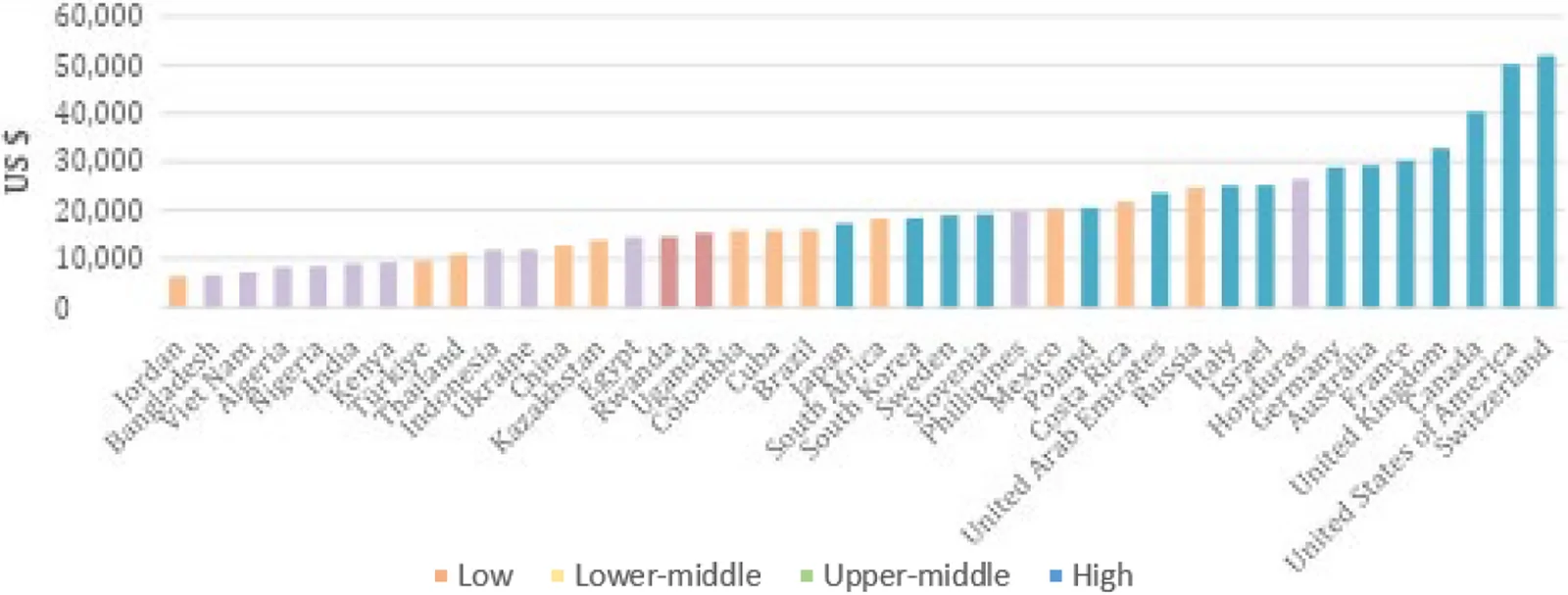
National average direct costs (US$) between the ages of 12-65 years

The projected population-level healthcare costs for managing dental caries from age 12 through 65 demonstrate substantial variation across the 40 countries analysed (Table 2). Total direct costs range dramatically, from US$372.8 million in Slovenia to US$182.5 billion in China, reflecting differences primarily driven by population size; China accounts for the largest national cohort studied, while Slovenia represents the smallest. When assessing costs on a per-person basis, Switzerland exhibits the highest estimate at US$51,932, whereas Jordan shows the lowest at US$6,339 (Fig. 3). These absolute figures should be interpreted as indicative orders of magnitude rather than precise, directly comparable budget estimates across countries, given underlying differences in unit costs and price levels. The primary focus of the analysis is therefore on within‑country differences across income quintiles, and on how these socioeconomic gradients manifest in diverse national contexts.

**Table 2:** Longitudinal direct costs of caries from 12 to 65 years of age by income quintile and population-level (US$)

Country	Highest income quintile	2nd highest income quintile	Middle income quintile	2nd lowest income quintile	Lowest income quintile	Total population-level
*Algeria*	950,791,239	1,061,020,191	906,795,968	886,513,455	1,014,840,671	4,819,961,524
*Australia*	1,301,560,453	1,553,145,191	1,417,625,189	1,438,338,095	1,666,012,427	7,376,681,355
*Bangladesh*	2,797,718,156	3,488,799,999	3,353,006,979	3,618,415,901	4,380,070,377	17,638,011,412
*Brazil*	5,182,848,053	6,343,925,860	6,412,786,773	8,704,546,823	10,538,822,271	37,182,929,780
*Canada*	2,207,404,682	2,780,480,819	2,742,696,902	2,958,503,584	3,491,987,417	14,181,073,404
*China*	29,550,965,652	36,606,046,276	35,170,991,238	37,302,187,937	43,948,407,221	182,578,598,324
*Colombia*	1,842,849,949	2,181,155,647	1,980,620,224	2,400,473,118	2,925,944,072	11,331,043,010
*Costa Rica*	230,466,640	273,569,857	260,321,073	334,962,170	401,773,497	1,501,093,237
*Cuba*	287,741,279	350,660,339	339,009,339	406,603,775	487,235,351	1,871,250,083
*Egypt*	3,666,832,185	4,279,268,876	3,826,819,771	3,800,844,724	4,331,935,752	19,905,701,308
*France*	3,444,420,670	4,209,775,993	4,043,997,000	4,339,009,118	5,048,373,442	21,085,576,223
*Germany*	3,225,830,758	4,018,846,625	3,751,943,652	3,663,943,893	4,289,315,396	18,949,880,324
*Honduras*	677,810,982	763,441,008	682,357,557	905,334,069	1,089,369,680	4,118,313,296
*India*	28,147,225,749	33,891,500,095	32,163,954,360	35,032,921,973	40,968,301,002	170,203,903,179
*Indonesia*	8,581,796,818	9,440,261,883	7,599,658,710	7,529,944,006	8,858,469,657	42,010,131,074
*Israel*	455,230,887	548,806,739	528,447,172	601,436,156	708,171,588	2,842,092,542
*Italy*	1,987,604,571	2,465,674,302	2,378,191,028	2,583,018,541	3,059,087,629	12,473,576,071
*Japan*	3,368,648,997	4,098,492,344	3,688,738,112	3,523,487,331	4,177,373,772	18,856,740,556
*Jordan*	145,089,246	179,892,907	174,989,084	189,076,527	222,536,890	911,584,654
*Kazakhstan*	451,550,926	521,829,317	475,276,976	500,992,296	576,276,627	2,525,926,142
*Kenya*	802,259,872	1,041,666,996	1,044,441,052	1,105,833,449	1,306,529,688	5,300,731,057
*Mexico*	6,004,829,587	6,865,401,824	6,156,418,131	7,397,912,144	8,775,529,351	35,200,091,037
*Nigeria*	3,162,660,369	3,582,455,655	3,142,589,943	3,533,080,254	4,169,971,124	17,590,757,345
*The Philippines*	6,192,033,019	6,702,600,933	5,448,957,538	5,762,264,649	6,799,432,421	30,905,288,560
*Poland*	1,134,705,315	1,344,600,734	1,284,745,995	1,380,661,385	1,558,950,233	6,703,663,662
*Russia*	3,264,059,960	4,149,047,233	4,344,839,985	5,430,051,102	6,429,601,520	23,617,599,800
*Rwanda*	527,016,596	601,477,256	522,293,329	595,948,988	717,225,031	2,963,961,200
*Slovenia*	48,383,297	61,250,669	62,833,539	67,605,191	77,755,524	317,828,220
*South Africa*	1,616,523,981	2,007,116,722	1,966,706,842	2,529,131,302	3,097,928,097	11,217,406,944
*South Korea*	1,716,677,804	2,113,494,390	2,093,241,272	2,368,260,859	2,746,881,700	11,038,556,025
*Sweden*	253,580,873	329,150,554	336,610,713	361,491,258	425,394,311	1,706,227,709
*Switzerland*	573,612,406	755,680,771	783,723,419	859,213,257	1,020,723,643	3,992,953,496
*Thailand*	1,496,732,166	1,771,548,418	1,636,058,796	1,720,704,398	1,979,174,964	8,604,218,742
*Türkiye*	1,656,747,843	2,004,000,814	1,918,183,194	2,349,974,087	2,816,726,036	10,745,631,974
*Uganda*	1,503,166,454	1,772,712,656	1,595,736,293	1,776,083,079	2,136,814,751	8,784,513,233
*Ukraine*	652,984,621	768,803,412	731,399,046	768,707,974	862,897,222	3,784,792,275
*UAE*	249,928,670	308,193,154	306,406,120	329,421,887	378,859,125	1,572,808,956
*UK*	4,071,877,948	5,040,266,992	4,778,129,491	4,895,635,989	5,728,910,752	24,514,821,172
*US*	27,825,473,696	33,640,461,153	31,748,668,009	34,937,192,524	40,658,650,917	168,810,446,299
*Viet Nam*	1,414,754,321	1,654,429,462	1,522,946,870	1,671,249,797	1,942,903,715	8,206,284,165

Analysis by income quintile reveals that individuals in the lowest income groups consistently incur statistically higher per-person costs than those in the highest income group (*p* < 0.05), as shown in Table 3. This difference is primarily driven by greater baseline caries prevalence and more rapid disease progression among lower income individuals. On average, direct dental costs are approximately 50% higher for the lowest income quintile compared to the highest. These findings persist even though the modelling accounted for the greater likelihood that individuals in the lowest income quintile would receive only an extraction and a less optimal, lower-cost replacement, while those in the highest income quintile were assumed to have a greater likelihood of receiving an implant after extraction.

**Table 3:** Direct per-person cost of caries between 12–65 years by income quintile (US$)

Country	Highest income quintile	2nd highest income quintile	Middle income quintile	2nd lowest income quintile	Lowest income quintile
*Algeria*	8,143	9,087	7,766	7,592	8,691
*Australia*	25,786	30,770	28,085	28,496	33,006
*Bangladesh*	5,251	6,549	6,294	6,792	8,222
*Brazil*	11,130	13,624	13,772	18,693	22,633
*Canada*	31,362	39,504	38,967	42,033	49,612
*China*	10,288	12,745	12,245	12,987	15,301
*Colombia*	12,834	15,190	13,793	16,717	20,377
*Costa Rica*	16,653	19,768	18,811	24,204	29,032
*Cuba*	12,156	14,814	14,322	17,178	20,584
*Egypt*	13,295	15,515	13,875	13,780	15,706
*France*	24,736	30,232	29,042	31,160	36,255
*Germany*	24,587	30,631	28,597	27,926	32,693
*Honduras*	21,716	24,459	21,862	29,005	34,902
*India*	7,430	8,946	8,490	9,247	10,814
*Indonesia*	12,108	13,319	10,722	10,624	12,498
*Israel*	20,163	24,307	23,406	26,638	31,366
*Italy*	19,962	24,763	23,885	25,942	30,723
*Japan*	15,426	18,768	16,892	16,135	19,130
*Jordan*	5,045	6,255	6,084	6,574	7,737
*Kazakhstan*	12,304	14,219	12,951	13,652	15,703
*Kenya*	6,968	9,048	9,072	9,605	11,349
*Mexico*	17,324	19,807	17,761	21,343	25,318
*Nigeria*	7,567	8,572	7,519	8,454	9,978
*The Philippines*	19,858	21,496	17,475	18,480	21,806
*Poland*	17,339	20,547	19,632	21,098	23,822
*Russia*	17,038	21,658	22,680	28,345	33,562
*Rwanda*	12,903	14,726	12,788	14,591	17,560
*Slovenia*	14,625	18,515	18,993	20,436	23,504
*South Africa*	13,203	16,393	16,063	20,657	25,302
*South Korea*	14,299	17,604	17,436	19,726	22,880
*Sweden*	14,082	18,279	18,693	20,075	23,624
*Switzerland*	37,301	49,141	50,965	55,874	66,377
*Thailand*	9,427	11,158	10,304	10,837	12,465
*Türkiye*	7,422	8,977	8,593	10,527	12,618
*Uganda*	13,110	15,461	13,917	15,490	18,636
*Ukraine*	10,258	12,078	11,490	12,076	13,556
*UAE*	18,740	23,109	22,975	24,700	28,407
*UK*	27,222	33,696	31,944	32,729	38,300
*US*	41,262	49,884	47,079	51,807	60,291
*Viet Nam*	6,209	7,261	6,684	7,335	8,527

**Fig. 4:**
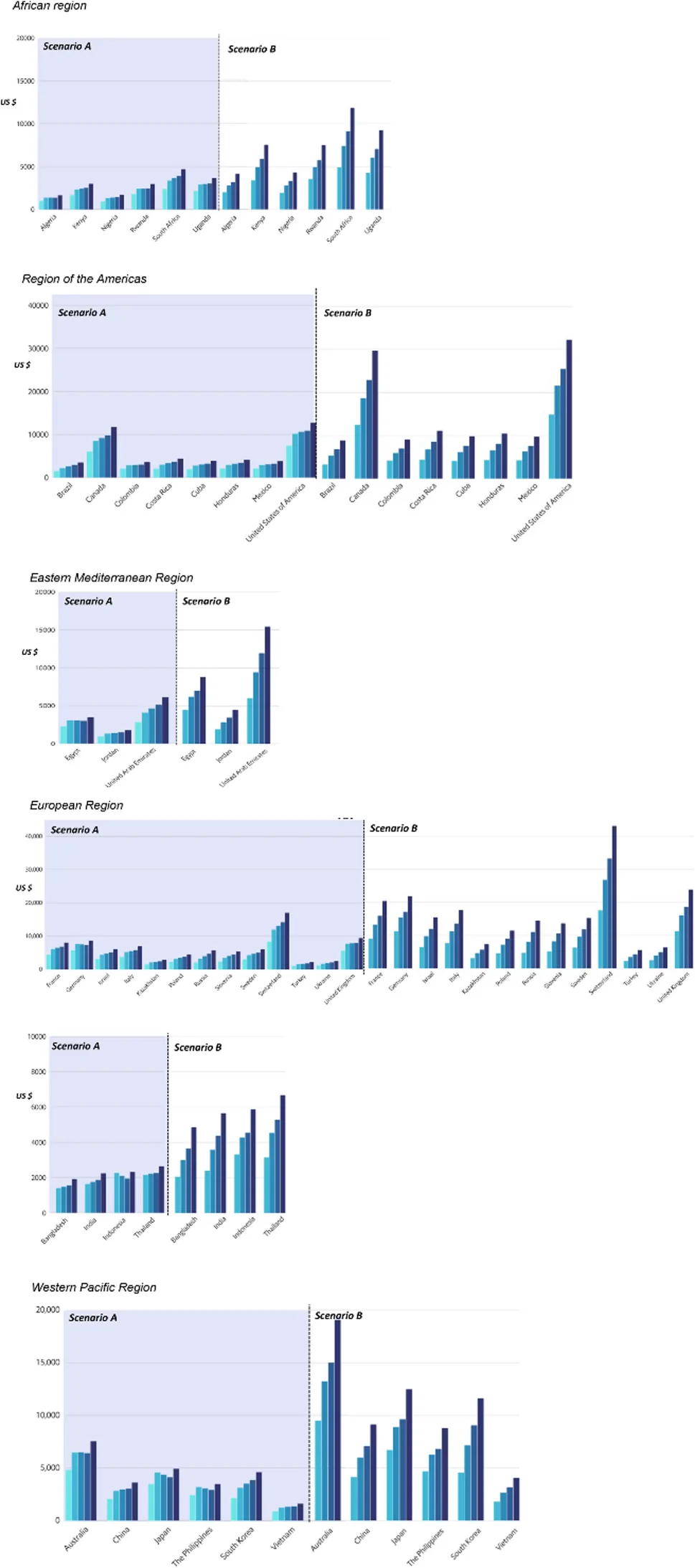
Decrease in per person costs (US$) after uniform application of non-targeted interventions lowering the progression rate by 30% (scenario A) and a levelling up approach to reduce caries progression (scenario B)

The impact of preventive interventions was explored through two modelled scenarios. Scenario A applied a uniform 30% reduction in annual caries progression rates across all income groups, reflecting potential effects of upstream and downstream prevention efforts. This conservative, intervention-agnostic estimate yielded the greatest per-person cost reductions in the lowest income quintile across all countries. In scenario B, a proportionate universalism approach was applied, where preventive and management interventions were proportionate to the degree of need across income quintiles. In this scenario, an annual caries progression rate of + 0.07 that originally pertained to the highest income group was applied across all quintiles, resulting in per-person cost reductions ranging from US$4,475 in Jordan to US$43,106 in Switzerland (Fig. 4).

To assess the robustness of the model outputs to key assumptions, we conducted two deterministic sensitivity analyses. In sensitivity analysis A, we increased the discount rate from 3.5% (base case) to 5%. This produced the same cost gradient across deprivation quintiles as in the base case, although absolute costs were lower due to the higher discount rate applied. In sensitivity analysis B, we varied treatment cost inputs by ± 10% around their base‑case values to reflect uncertainty in unit cost estimates. This resulted in a corresponding ± 10% change in the estimated economic burden within each deprivation quintile and country, but did not alter the relative ranking of quintiles or the overall magnitude of inequalities. Full results for both sensitivity analyses are presented in Supplementary file 1.

## Discussion

Building upon our previous six-country model of income-stratified caries costs and prevention scenarios (in Brazil, France, Germany, Indonesia, Italy, and the UK), this study extends its conceptual and economic framework to 40 countries. We estimated the direct, longitudinal costs of caries in permanent teeth among 12-year-olds, examining variation across socioeconomic strata and national contexts. Our analysis reveals that individuals in the lowest income groups face treatment costs approximately 50% higher than their wealthier peers, a disparity driven by limited access to preventive care, higher baseline rates of caries, and more rapid progression of tooth decay. These inequities persist even when accounting for lower-cost and often suboptimal treatments or inappropriate treatment options, such as tooth extraction when a restorative procedure may be more appropriate, are more frequently provided to disadvantaged groups.

These socioeconomic disparities exist alongside significant variations between countries, reflecting both epidemiological and economic drivers. Nine of the ten highest per-person cost estimates are in high-income countries, reflecting higher unit prices, while Honduras emerges as an exception due to its particularly high baseline caries burden. Baseline DMFT (dentine threshold) values span wide extremes, from 0.4 (Kenya) to 3.7 (Honduras), underscoring the heterogeneity that policymakers must address. These variations highlight the importance of tailoring prevention and care strategies to local contexts, accounting for both resource availability and population-level risk profiles. Recent work across 11 Latin American Countries with the International Caries Detection and Assessment System (ICDAS) adapted for Early Childhood Caries has provided an epidemiological tool for monitoring initial, moderate and extensive caries to assess preventive interventions prior to children reaching age 12 years [30].

A critical challenge emerges from these findings: access to effective and affordable preventive interventions remains severely limited in many countries, compounding health and economic inequalities [31]. Strategies such as twice daily brushing with a fluoride-containing toothpaste, fluoride varnish and gel application, fissure sealants, Silver Diamine Fluoride (SDF) and sugar intake reduction have shown considerable impact and cost-effectiveness in lowering caries prevalence [25, 27, 32, 33]. Population-level measures, including community water fluoridation, oral health education and SSB taxes, further complement these approaches and especially benefit vulnerable communities [28, 34, 35]. However, implementation of these low-cost, high-efficacy interventions is still not universal, particularly among low-income populations.

These challenges are precisely what high-level policy frameworks aim to address. The WHO Global strategy and action plan on oral health 2023–2030 and the recent Bangkok Declaration: No Health Without Oral Health (endorsed by WHO Member States in November 2024), provide policy frameworks for tackling these challenges [31]. The Bangkok Declaration in particular, calls for strengthening primary care-led, prevention-oriented systems by embedding oral health within the universal health coverage agenda. Achieving this vision requires a multipronged strategy across the life course, integrating early interventions, accessible clinical treatments, and essential upstream policies such as community water fluoridation and SSB taxes. Despite the proven effectiveness of these interventions, their adoption and implementation remain inconsistent across countries. Among the 40 countries examined, only half have national SSB taxes, and less than 7% of the global population benefits from fluoridated water supplies [35, 36]. These disparities underscore the urgent need for stronger policy adoption and comprehensive health system reforms to reduce oral health inequities and advance outcomes globally.

A further barrier to policy and service reform is the lack of robust, current surveillance data in many countries. Although studies demonstrate efficacy for measures like water fluoridation (with a 26% caries reduction in permanent teeth) and fluoridated toothpaste (24% reduction), few countries collect adequate real-world, national-level data to assess cumulative impact or monitor progress [32, 37]. For instance, Honduras and Vietnam’s last national oral health surveys dates to 1997 and 1999 respectively, highlighting the necessity of updated surveillance to guide effective planning [17, 38]. The lack of recent data makes it difficult to track progress, assess inequalities, or design evidence-based interventions. Strengthening surveillance systems and integrating oral health indicators into national health surveys would enable countries to better estimate their burden of disease and align resources with need [31].

The limitations of our model stem from challenges in sourcing appropriate, consistent data inputs across 40 heterogeneous countries. Expanding this research to a larger and more diverse group of settings inevitably introduces considerable variability in epidemiology, health systems and cost structures, and increases reliance on estimated inputs for some parameters. Baseline DMFT scores were derived from national oral health surveys spanning more than two decades (1997–2021), introducing temporal heterogeneity. This is a relevant concern, given the general decline in DMFT in high-income countries over recent decades, contrasted by stagnating or upward trends in middle- and low-income settings [39]. In addition, caries prevalence stratified by socioeconomic groups was unavailable for all countries, requiring extrapolation based on limited evidence. Annual progression rates of dental caries across income groups remain poorly documented, necessitating assumptions informed by existing literature [24]. While it is established that lower income populations are less likely to receive restorative treatments and more likely to undergo extractions, precise treatment rate differences between income groups are not well quantified. Public sector cost data for caries care are scarce; thus, private sector costs were used, drawing from online sources and expert input, but these estimates exhibit substantial variability and reflect country‑specific price levels rather than a harmonised international cost base. Consequently, the absolute monetary estimates presented here should be interpreted as indicative orders of magnitude rather than precise, directly comparable budget figures across countries. Notably, probabilities for tooth health transitions were sourced from a single New Zealand cohort and applied universally, which was necessary due to the lack of longitudinal data of this nature, however, it may limit the generalisability of transition dynamics across diverse country contexts. Taken together, these data gaps underscore the need for improved longitudinal and stratified data collection to refine economic burden models and enhance their policy relevance. At the same time, our findings are most appropriately interpreted in terms of within‑country socioeconomic gradients in the economic burden of caries, and in how these gradients consistently disadvantage lower‑income groups across a wide range of health‑system and epidemiological settings.

## Conclusion

Our analysis of 40 countries highlights the disproportionate economic burden of dental caries on the most socioeconomically disadvantaged populations, driven by higher disease prevalence, faster progression, and limited access to preventive care. Early, targeted preventive interventions—such as twice daily bushing with fluoridated toothpaste, water fluoridation, oral health education, and school-based programmes—delivered within a life-course, equity-driven framework, are crucial to reducing these inequities and advancing global oral health.

Achieving meaningful progress will require stronger intersectoral collaboration, ensuring that every encounter across health, education, and community settings contributes to oral disease prevention and health promotion. Embedding core oral health competencies into the curricula for all healthcare and allied professionals is essential, equipping the workforce to identify, prevent, and manage dental caries holistically and collaboratively.

A further priority is the development and international advocacy of a minimal oral health dataset, to be agreed upon and adopted for population surveys. Establishing this standardised dataset will strengthen health surveillance, bolster research, and inform targeted interventions.

Finally, integrating dental care into universal health coverage, reinforcing health surveillance systems, and advancing the policy reforms called for by recent WHO frameworks will be pivotal. By acting on these priorities, countries can reduce the health and societal impact of dental caries and advance the goal of oral health equity worldwide.

## Supplementary Information

Supplementary Material 1.

## Data Availability

The dataset generated during this analysis is available from the corresponding author upon reasonable request.

## Abbreviations

CHEERS: Consolidated Health Economic Evaluation Reporting Standards
DMFT: Decayed, Missing or Filled Teeth
DT: Decayed teeth
FT: Filled teeth
ICDAS: International Caries Detection and Assessment System
MT: Missing teeth
SDF: Silver Diamine Fluoride
SSB: Sugar-sweetened beverage
UK: United Kingdom
US: United States of America
UAE: United Arab Emirates
WHO: World Health Organization
WPP: World Population Prospects
