# Supplementary material for: Socioeconomic inequalities and the economic burden of dental caries: a longitudinal simulation analysis across forty countries
Source: BMC Public Health. 2026 Jul 24;26:3009. doi: 10.1186/s12889-026-28363-4 (PMC13681945; doi:10.1186/s12889-026-28363-4)
Supplement: Supplementary file 1 [file 12889_2026_28363_MOESM1_ESM.docx]

**Supplementary File 1**

**Supplementary Table 1. DMFT scores by income quintile and country**

|  | **Highest income quintile** | **2nd highest income quintile** | **Middle income quintile** | **2nd lowest income quintile** | **Lowest income quintile** | **Year DMFT data was collected** |
| --- | --- | --- | --- | --- | --- | --- |
| **Algeria** | 2.08 | 2.6 | 2.6 | 3.21 | 3.52 | 2013 |
| **Australia** | 0.88 | 1.1 | 1.1 | 1.6 | 1.85 | 2009 |
| **Bangladesh** | 0.8 | 1 | 1 | 1.43 | 1.64 | 2000 |
| **Brazil** | 1.66 | 2.07 | 2.07 | 4.27 | 5.37 | 2010 |
| **Canada** | 0.8 | 1 | 1 | 1.37 | 1.56 | 2009 |
| **China** | 0.72 | 0.9 | 0.9 | 1.35 | 1.58 | 2016 |
| **Colombia** | 1.2 | 1.5 | 1.5 | 3.19 | 4.03 | 2014 |
| **Costa Rica** | 2 | 2.5 | 2.5 | 4.65 | 5.73 | 2006 |
| **Cuba** | 1.2 | 1.5 | 1.5 | 2.76 | 3.39 | 2005 |
| **Egypt** | 1.04 | 1.3 | 1.3 | 1.79 | 2.04 | 2016 |
| **France** | 0.98 | 1.23 | 1.23 | 1.77 | 2.03 | 2006 |
| **Germany** | 0.4 | 0.5 | 0.5 | 0.69 | 0.78 | 2016 |
| **Honduras** | 2.96 | 3.7 | 3.7 | 7.07 | 8.75 | 2008 |
| **India** | 1.28 | 1.6 | 1.6 | 2.25 | 2.58 | 2012 |
| **Indonesia** | 1.52 | 1.9 | 1.9 | 2.99 | 3.53 | 2018 |
| **Israel** | 1.36 | 1.7 | 1.7 | 2.67 | 3.16 | 2002 |
| **Italy** | 0.87 | 1.09 | 1.09 | 1.62 | 1.88 | 2005 |
| **Japan** | 0.48 | 0.6 | 0.6 | 0.85 | 0.97 | 2021 |
| **Jordan** | 0.88 | 1.1 | 1.1 | 1.58 | 1.82 | 2005 |
| **Kazakhstan** | 1.98 | 2.48 | 2.48 | 3.2 | 3.55 | 2015 |
| **Kenya** | 0.32 | 0.4 | 0.4 | 0.64 | 0.76 | 2015 |
| **Mexico** | 2.08 | 2.6 | 2.6 | 4.57 | 5.55 | 2018 |
| **Nigeria** | 1.44 | 1.8 | 1.8 | 2.67 | 3.1 | 2013 |
| **Philippines** | 2 | 2.5 | 2.5 | 4.16 | 4.99 | 2018 |
| **Poland** | 1.92 | 2.4 | 2.4 | 3.04 | 3.36 | 2019 |
| **Russia** | 2 | 2.5 | 2.5 | 3.7 | 4.31 | 2008 |
| **Rwanda** | 1.67 | 2.09 | 2.09 | 3.69 | 4.48 | 2016 |
| **Slovenia** | 1.2 | 1.5 | 1.5 | 1.69 | 1.79 | 2017 |
| **South Africa** | 0.88 | 1.1 | 1.1 | 2.63 | 3.4 | 2017 |
| **South Korea** | 1.44 | 1.8 | 1.8 | 2.54 | 2.91 | 2012 |
| **Sweden** | 0.56 | 0.7 | 0.7 | 0.92 | 1.02 | 2021 |
| **Switzerland** | 0.48 | 0.6 | 0.6 | 0.86 | 0.99 | 2021 |
| **Thailand** | 1.12 | 1.4 | 1.4 | 2.07 | 2.4 | 2017 |
| **Türkiye** | 1.52 | 1.9 | 1.9 | 3.39 | 4.14 | 2005 |
| **Uganda** | 1.12 | 1.4 | 1.4 | 2.42 | 2.93 | 2018 |
| **Ukraine** | 2.24 | 2.8 | 2.8 | 3.28 | 3.52 | 2008 |
| **UAE** | 1.44 | 1.8 | 1.8 | 2.15 | 2.33 | 2013 |
| **UK** | 0.64 | 0.8 | 0.8 | 1.12 | 1.28 | 2017 |
| **US** | 0.96 | 1.2 | 1.2 | 2.02 | 2.43 | 2004 |
| **Viet Nam** | 1.52 | 1.9 | 1.9 | 2.88 | 3.37 | 2001 |

**Supplementary Table 2: Assumptions used in the distribution of DMFT progression between age cohorts across income quintiles (% of total change in DMFT score attributed to decayed teeth, filled teeth and missing teeth vs middle income quintile)**

|  | **12 years** | **13-18 years** | **19-26 years** | **27-32 years** | **33-38 years** | **39-65 years** |
| --- | --- | --- | --- | --- | --- | --- |
| ***Highest income vs middle income*** | | | | | | |
| **Decayed** | Baseline | 3.57% | 51.7% | -35% | -43% | 24% |
| **Filled** | Baseline | 96.4% | 42.9% | 110% | 68% | 33% |
| **Missing** | Baseline | 0% | 5.3% | 25% | 75% | 43% |
| ***Second highest income vs middle income*** | | | | | | |
| **Decayed** | Baseline | 5.7% | 56.7% | -37% | 47.5% | 26.3% |
| **Filled** | Baseline | 94.2% | 35.2% | 107% | 62.5% | 27.7% |
| **Missing** | Baseline | 0% | 8.1% | 30% | 85% | 46% |
| ***Middle income*** | | | | | | |
| **Decayed** | Baseline | 7.1% | 60.7% | -40% | -50% | 27.7% |
| **Filled** | Baseline | 92.8% | 28.5% | 100% | 50% | 22.2% |
| **Missing** | Baseline | 0% | 10.7% | 40% | 100% | 50% |
| ***Second lowest income vs middle income*** | | | | | | |
| **Decayed** | Baseline | 10% | 64.3% | -47.2% | -60% | 30% |
| **Filled** | Baseline | 90% | 21.6% | 92.2% | 45% | 10% |
| **Missing** | Baseline | 0% | 15% | 55% | 115% | 60% |
| ***Lowest income vs middle income*** | | | | | | |
| **Decayed** | Baseline | 14.3% | 68% | -55% | -70% | 31.8% |
| **Filled** | Baseline | 85.7% | 12.7% | 75% | 40% | 3.2% |
| **Missing** | Baseline | 0% | 19.3% | 80% | 130% | 65% |

**Supplementary Table 3: Costs associated with dental caries management per tooth (2025, in US$, based on currency conversion rates on 15/01/2025)**

|  | **Filling** | **Root canal** | **Crown** | **Extraction** | **Implant** | **Alternative replacement*** |
| --- | --- | --- | --- | --- | --- | --- |
| **Algeria** | 28.8 | 98.6 | 219 | 18.3 | 600.8 | 90.1 |
| **Australia** | 119.2 | 699.3 | 1002.3 | 127.428 | 2,651.7 | 397.8 |
| **Bangladesh** | 29.7 | 101 | 96.35 | 22.55 | 512.5 | 76.9 |
| **Brazil** | 64 | 178.5 | 674 | 39.6 | 432 | 64.8 |
| **Canada** | 210.45 | 594.09 | 776.3 | 131.1 | 2,972.9 | 445.9 |
| **China** | 57.7 | 228.8 | 407.5 | 61.3 | 1,019.6 | 152.9 |
| **Colombia** | 57.5 | 149.5 | 241.5 | 46 | 1,150 | 172.5 |
| **Costa Rica** | 78.85 | 228 | 404.7 | 63.7 | 868.3 | 130.2 |
| **Cuba** | 65.1 | 192.9 | 411.1 | 49.3 | 852.6 | 127.9 |
| **Egypt** | 47.6 | 337.6 | 429.2 | 38.6 | 1,104.6 | 165.7 |
| **France** | 139.1 | 618 | 861.4 | 87.5 | 2,060 | 309 |
| **Germany** | 139.2 | 697 | 1081 | 40.9 | 3,299.5 | 494.9 |
| **Honduras** | 65.1 | 192.9 | 411.1 | 49.3 | 852.6 | 127.9 |
| **India** | 33 | 110 | 159.5 | 33 | 495 | 74.25 |
| **Indonesia** | 27.5 | 121.7 | 158.6 | 28.9 | 1,043.1 | 156.5 |
| **Israel** | 108.3 | 378.5 | 947.9 | 121.7 | 1,353.7 | 203.1 |
| **Italy** | 126.2 | 360.5 | 439.1 | 62.6 | 1,957 | 293.6 |
| **Japan** | 70.7 | 280.6 | 841.7 | 104.8 | 2,167.1 | 325.1 |
| **Jordan** | 30.1 | 99.9 | 167.7 | 21.3 | 441.3 | 66.2 |
| **Kazakhstan** | 45 | 144 | 243 | 32.4 | 666 | 99.9 |
| **Kenya** | 34.65 | 154 | 216.6 | 30.8 | 635.25 | 95.3 |
| **Mexico** | 60 | 216 | 324 | 48 | 960 | 144 |
| **Nigeria** | 20.8 | 96 | 121.6 | 42.1 | 566.4 | 84.9 |
| **Philippines** | 42.5 | 178.5 | 527 | 51 | 1,275 | 191.3 |
| **Poland** | 76.8 | 384 | 420 | 60 | 820.8 | 123.1 |
| **Russia** | 97 | 139.68 | 291 | 48.5 | 507.6 | 76.1 |
| **Rwanda** | 47.3 | 101.25 | 162 | 17.6 | 1,012.5 | 151.9 |
| **Slovenia** | 103.5 | 327.5 | 444.2 | 51.5 | 875.5 | 131.3 |
| **South Africa** | 62.6 | 165.6 | 344.3 | 42.9 | 900 | 135 |
| **South Korea** | 85 | 334.4 | 340.3 | 87 | 884 | 132.6 |
| **Sweden** | 111.1 | 379.4 | 571.7 | 79.2 | 1,400 | 210 |
| **Switzerland** | 313.4 | 926.1 | 1,506.9 | 262.9 | 3,864.1 | 579.6 |
| **Thailand** | 40 | 245 | 335 | 37 | 730 | 109.5 |
| **Türkiye** | 37.4 | 99.9 | 97.2 | 37.2 | 494.7 | 74.2 |
| **Uganda** | 47.3 | 101.3 | 162 | 17.6 | 1,012.5 | 151.9 |
| **Ukraine** | 39 | 165 | 206 | 42 | 405 | 60.8 |
| **UAE** | 114.8 | 386.9 | 594 | 128.3 | 1,154.3 | 173.1 |
| **UK** | 150.7 | 704.6 | 876.8 | 163.3 | 3,062.2 | 459.3 |
| **US** | 205 | 1200 | 1,300 | 154.25 | 3,500 | 525 |
| **Viet Nam** | 25.4 | 115.1 | 195 | 25.4 | 405.6 | 60.8 |

**Dental bridges or single-tooth removable partial dentures are alternatives to dental implants in replacing a missing tooth. We included an alternative replacement option in the analysis to account for treatment options that cost lower than a dental implant*

**Supplementary Table 4: Provision of root canals and crown across income groups (%)**

|  | Root canal | Crown |
| --- | --- | --- |
| Highest income | 18 | 7.2 |
| 2^nd^ highest income | 12.5 | 3.75 |
| Middle income | 9 | .18 |
| 2nd lowest income | 5.5 | 0 |
| Lowest income | 1.5 | 0 |

**Supplementary Table 5:** **Provision of replacements across income groups (%)**

|  | Receive single implant^ | Receive cheaper alternative replacement* |
| --- | --- | --- |
| Highest income | 100 | 0 |
| 2^nd^ highest income | 60 | 40 |
| Middle income | 20 | 80 |
| 2nd lowest income | 0 | 100 |
| Lowest income | 0 | 100 |

^Assumes patients are suitable candidates for the implants (have good bone support)

*The cheaper replacement in each country was 15% of the cost of the single implant

**Supplementary Table 6. Sensitivity analysis b: Direct costs of dental caries from 12 to 65 years of age (US$) (range if costs were 10% lower to 10% higher than treatment costs applied in baseline analysis)**

| **Country** | **Highest income quintile** | **2nd highest income quintile** | **Middle income quintile** | **2nd lowest income quintile** | **Lowest income quintile** |
| --- | --- | --- | --- | --- | --- |
| *Algeria* | 7,329 - 8,957 | 8,178 - 9,996 | 6,989 - 8,543 | 6,833 - 8,351 | 7,822 - 9,560 |
| *Australia* | 23,207 - 28,365 | 27,693 - 33,847 | 25,277 - 30,894 | 25,646 - 31,346 | 29,705 - 36,307 |
| *Bangladesh* | 4,726 - 5,776 | 5,894 - 7,204 | 5,665 - 6,923 | 6,113 - 7,471 | 7,400 - 9,044 |
| *Brazil* | 10,017 - 12,243 | 12,262 - 14,986 | 12,395 - 15,149 | 16,824 - 20,562 | 20,370 - 24,896 |
| *Canada* | 28,226 - 34,498 | 35,554 - 43,454 | 35,070 - 42,864 | 37,830 - 46,236 | 44,651 - 54,573 |
| *China* | 9,259 - 11,317 | 11,471 - 14,020 | 11,021 - 13,470 | 11,688 - 14,286 | 13,771 - 16,831 |
| *Colombia* | 11,551 - 14,117 | 13,671 - 16,709 | 12,414 - 15,172 | 15,045 - 18,389 | 18,339 - 22,415 |
| *Costa Rica* | 14,988 - 18,318 | 17,791 - 21,745 | 16,930 - 20,692 | 21,784 - 26,624 | 26,129 - 31,935 |
| *Cuba* | 10,940 - 13,372 | 13,333 - 16,295 | 12,890 - 15,754 | 15,460 - 18,896 | 18,526 - 22,642 |
| *Egypt* | 11,966 - 14,625 | 13,964 - 17,067 | 12,488 - 15,263 | 12,402 - 15,158 | 14,135 - 17,277 |
| *France* | 22,262 - 27,210 | 27,209 - 32,255 | 26,138 - 31,946 | 28,044 - 34,276 | 32,630 - 39,881 |
| *Germany* | 22,128 - 27,046 | 27,568 - 33,694 | 25,737 - 31,457 | 25,133 - 30,717 | 29,424 - 35,962 |
| *Honduras* | 19,544 - 23,888 | 22,013 - 26,905 | 19,676 - 24,048 | 26,105 - 31,906 | 31,412 - 38,392 |
| *India* | 6,687 - 8,173 | 8,051 - 9,841 | 7,641 - 9,339 | 8,322 - 10,172 | 9,733 - 11,895 |
| *Indonesia* | 10,897 - 13,319 | 11,987 - 14,651 | 9,650 - 11,794 | 9,562 - 11,686 | 11,248 - 13,748 |
| *Israel* | 18,147 - 22,179 | 21,876 - 26,738 | 21,065 - 25,747 | 23,974 - 29,302 | 28,230 - 34,503 |
| *Italy* | 17,966 - 21,958 | 22,287 - 27,239 | 21,497 - 26,274 | 23,348 - 28,536 | 27,651 - 33,795 |
| *Japan* | 13,884 - 16,969 | 16,891 - 20,645 | 15,203 - 18,581 | 14,522 - 17,749 | 17,217 - 21,043 |
| *Jordan* | 4,541 - 5,550 | 5,630 - 6,881 | 5,476 - 6,692 | 5,917 - 7,231 | 6,963 - 8,511 |
| *Kazakhstan* | 11,074 - 13,534 | 12,797 - 15,641 | 11,656 - 14,246 | 12,287 - 15,017 | 14,133 - 17,273 |
| *Kenya* | 6,271 - 7,665 | 8,143 - 9,953 | 8,165 - 9,979 | 8,645 - 10,566 | 10,214 - 12,484 |
| *Mexico* | 15,592 - 19,056 | 17,826 - 21,788 | 15,985 - 19,537 | 19,208 - 23,477 | 22,786 - 27,850 |
| *Nigeria* | 6,810 - 8,324 | 7,715 - 9,429 | 6,767 - 8,271 | 7,609 - 9,299 | 8,980 - 10,976 |
| *The Philippines* | 17,872 - 21,844 | 19,346 - 23,646 | 15,728 - 19,223 | 16,632 - 20,328 | 19,625 - 23,987 |
| *Poland* | 15,605 - 19,073 | 18,492 - 22,602 | 17,669 - 21,595 | 18,988 - 23,208 | 21,440 - 26,204 |
| *Russia* | 15,334 - 18,742 | 19,492 - 23,824 | 20,412 - 24,948 | 25,511 - 31,180 | 30,206 - 36,918 |
| *Rwanda* | 11,613 - 14,193 | 13,253 - 16,199 | 11509 - 14,067 | 13,132 - 16,050 | 15,804 - 19,316 |
| *Slovenia* | 13,163 - 16,088 | 16,664 - 20,367 | 17,094 - 20,892 | 18,392 - 22,480 | 21,154 - 25,854 |
| *South Africa* | 11,883 - 14,523 | 14,754 - 18,032 | 14,457 - 17,669 | 18,591 - 22,723 | 22,772 - 27,832 |
| *South Korea* | 12,869 - 15,729 | 15,844 - 19,364 | 15,692 - 19,180 | 20,592 - 25,168+P72:U74 | 20,592 - 25,168 |
| *Sweden* | 12,674 - 15,490 | 16,451 - 20,107 | 16,824 - 20,562 | 18,068 - 22,083 | 21,262 - 25,986 |
| *Switzerland* | 33,571 - 41,031 | 44,227 - 54,055 | 45,869 - 56,062 | 50,287 - 61,461 | 59,739 - 73,015 |
| *Thailand* | 8,484 - 10,370 | 10,042 - 12,274 | 9,274 - 11,334 | 9,753 - 11,921 | 11,219 - 13,712 |
| *Türkiye* | 6,680 - 8,164 | 8,079 - 9,875 | 7,734 - 9,452 | 9,474 - 9,452 | 11,356 - 13,880 |
| *Uganda* | 11,799 - 14,421 | 13,915 - 17,007 | 12,525 - 15,309 | 13,941 - 17,039 | 16,772 - 20,500 |
| *Ukraine* | 9,232 - 11,284 | 10,870 - 13,286 | 10,341 - 12,639 | 10,868 - 13,284 | 12,200 - 14,912 |
| *UAE* | 16,866 - 20,614 | 20,798 - 25,420 | 20,678 - 25,273 | 22,230 - 27,170 | 25,566 - 31,248 |
| *UK* | 24,500 - 29,944 | 30,326 - 37,066 | 28,750 - 35,138 | 29,456 - 36,002 | 34,470 - 42,130 |
| *US* | 37,136 - 45,388 | 44,896 - 54,872 | 42,371 - 51,787 | 46,626 - 56,988 | 54,262 - 66,320 |
| *Viet Nam* | 5,588 - 6,830 | 6,535 - 7,987 | 6,016 - 7,352 | 6,602 - 8,069 | 7,674 - 9,380 |

**Supplementary Table 7: Consolidated Health Economic Evaluation Reporting Standards 2022 (CHEERS 2022) Checklist**

| **Topic** | **No.** | **Item** | **Location where item is reported** |
| --- | --- | --- | --- |
| **Title** |  |  |  |
|  | 1 | Identify the study as an economic evaluation and specify the interventions being compared. | page 1 |
| **Abstract** |  |  |  |
|  | 2 | Provide a structured summary that highlights context, key methods, results, and alternative analyses. | pages 1-2 |
| **Introduction** |  |  |  |
| **Background and objectives** | 3 | Give the context for the study, the study question, and its practical relevance for decision making in policy or practice. | pages 4-5 |
| **Methods** |  |  |  |
| **Health economic analysis plan** | 4 | Indicate whether a health economic analysis plan was developed and where available. | N/A |
| **Study population** | 5 | Describe characteristics of the study population (such as age range, demographics, socioeconomic, or clinical characteristics). | N/A |
| **Setting and location** | 6 | Provide relevant contextual information that may influence findings. | pages 4 and 9 |
| **Comparators** | 7 | Describe the interventions or strategies being compared and why chosen. | N/A |
| **Perspective** | 8 | State the perspective(s) adopted by the study and why chosen. | page 5-7 |
| **Time horizon** | 9 | State the time horizon for the study and why appropriate. | page 5-7 |
| **Discount rate** | 10 | Report the discount rate(s) and reason chosen. | page 12 |
| **Selection of outcomes** | 11 | Describe what outcomes were used as the measure(s) of benefit(s) and harm(s). | pages 5-12 |
| **Measurement of outcomes** | 12 | Describe how outcomes used to capture benefit(s) and harm(s) were measured. | pages 5-12 |
| **Valuation of outcomes** | 13 | Describe the population and methods used to measure and value outcomes. | pages 5-12 |
| **Measurement and valuation of resources and costs** | 14 | Describe how costs were valued. | Table 3 in the supplementary file |
| **Currency, price date, and conversion** | 15 | Report the dates of the estimated resource quantities and unit costs, plus the currency and year of conversion. | Table 3 in the supplementary file |
| **Rationale and description of model** | 16 | If modelling is used, describe in detail and why used. Report if the model is publicly available and where it can be accessed. | pages 5-12 |
| **Analytics and assumptions** | 17 | Describe any methods for analysing or statistically transforming data, any extrapolation methods, and approaches for validating any model used. | pages 5-12 |
| **Characterising heterogeneity** | 18 | Describe any methods used for estimating how the results of the study vary for subgroups. | pages 8-11 |
| **Characterising distributional effects** | 19 | Describe how impacts are distributed across different individuals or adjustments made to reflect priority populations. | pages 8-11 |
| **Characterising uncertainty** | 20 | Describe methods to characterise any sources of uncertainty in the analysis. | page 12 |
| **Approach to engagement with patients and others affected by the study** | 21 | Describe any approaches to engage patients or service recipients, the general public, communities, or stakeholders (such as clinicians or payers) in the design of the study. | page 9 |
| **Results** |  |  |  |
| **Study parameters** | 22 | Report all analytic inputs (such as values, ranges, references) including uncertainty or distributional assumptions. | pages 8-11 |
| **Summary of main results** | 23 | Report the mean values for the main categories of costs and outcomes of interest and summarise them in the most appropriate overall measure. | pages 12-17 |
| **Effect of uncertainty** | 24 | Describe how uncertainty about analytic judgments, inputs, or projections affect findings. Report the effect of choice of discount rate and time horizon, if applicable. | page 12 |
| **Effect of engagement with patients and others affected by the study** | 25 | Report on any difference patient/service recipient, general public, community, or stakeholder involvement made to the approach or findings of the study | page 9 |
| **Discussion** |  |  |  |
| **Study findings, limitations, generalisability, and current knowledge** | 26 | Report key findings, limitations, ethical or equity considerations not captured, and how these could affect patients, policy, or practice. | pages 21-24 |
| **Other relevant information** |  |  |  |
| **Source of funding** | 27 | Describe how the study was funded and any role of the funder in the identification, design, conduct, and reporting of the analysis | page 25 |
| **Conflicts of interest** | 28 | Report authors conflicts of interest according to journal or International Committee of Medical Journal Editors requirements. | page 26 |
